# Comprehensive Healthcare module: medical and pharmacy students’ shared learning experiences

**DOI:** 10.3402/meo.v19.25605

**Published:** 2014-10-17

**Authors:** Chai-Eng Tan, Aida Jaffar, Seng-Fah Tong, Majmin Sheikh Hamzah, Nabishah Mohamad

**Affiliations:** Department of Family Medicine, Faculty of Medicine, Universiti Kebangsaan Malaysia Medical Centre, Kuala Lumpur, Malaysia

**Keywords:** education, medical, undergraduate, inter-professional education, comprehensive healthcare, primary healthcare

## Abstract

**Introduction:**

The Comprehensive Healthcare (CHC) module was developed to introduce pre-clinical medical and pharmacy students to the concept of comprehensive healthcare. This study aims to explore their shared learning experiences within this module.

**Methodology:**

During this module, medical and pharmacy students conducted visits to patients’ homes and to related community-based organisations in small groups. They were required to write a reflective journal on their experiences regarding working with other professions as part of their module assessment. Highly scored reflective journals written by students from the 2011/2012 academic session were selected for analysis. Their shared learning experiences were identified via thematic analysis. We also analysed students’ feedback regarding the module.

**Results:**

Analysis of 25 selected reflective journals revealed several important themes: ‘Understanding of impact of illness and its relation to holistic care’, ‘Awareness of the role of various healthcare professions’ and ‘Generic or soft skills for inter-professional collaboration’. Although the primary objective of the module was to expose students to comprehensive healthcare, the students learnt skills required for future collaborative practice from their experiences.

**Discussion:**

The CHC module provided early clinical exposure to community-based health issues and incorporated some elements of inter-professional education. The students learnt about the roles of other healthcare professions and acquired soft skills required for future collaborative practice during this module.

An increasing number of professions and specialisations within the medical, health and social services have led to fragmentation of healthcare service. This could lead to healthcare providers losing the concept of seeing a patient as ‘a whole’ and compromising the quality of healthcare. Early exposure to the concept of comprehensive care among healthcare students can help them to appreciate the importance of a holistic approach in patient assessment and management. Various studies have demonstrated that early exposure to clinical and community-based healthcare had positive effects on the students in terms of learning, knowledge and skills (1).

Provision of comprehensive healthcare requires multi-disciplinary teamwork. Teamwork and collaboration among different healthcare professionals are important elements that should be taught to healthcare students (2). Inter-professional education (IPE) is defined by the Centre for Advancements of Inter-Professional Education (CAIPE) as ‘occasions when two or more professions learn with, from and an about each other to improve collaboration and quality of care’ (3). IPE fosters an inclusive view of ‘professionalism’, whereby members of the healthcare team develop healthy respect for one another, as well as working towards a patient-centred focus (2). IPE has potential to positively affect clinical outcomes (4). Systematic review of studies regarding the effect of IPE on clinical outcomes found evidence of improved communication between healthcare professionals as well as quality of healthcare services (5).

Recognising the potential of IPE, a Malaysian public medical school introduced elements of IPE into the comprehensive healthcare module for undergraduate medical and pharmacy students. The Comprehensive Healthcare (CHC) module was developed by an inter-professional group of educators from the medical faculty and implemented beginning the year 2007. It was a compulsory module for Year 2 undergraduate medical students and was offered as an elective for up to 50 Year 3 undergraduate pharmacy students. The main objective of this study was to explore the students’ perception on their shared learning experiences as well as benefits gained during this educational programme.

## Methodology

The CHC module was a 2-credit module. At the end of the module, students were expected to understand the concept of comprehensive healthcare and to apply it in the management of patients’ health problems.

Student-initiated hands-on sessions such as small-group discussions, visits to patients’ homes and community-based healthcare-related organisations were arranged to further enhance students’ understanding of the impact of the diseases to the patient, family and community. Small-group discussions were facilitated by academic staff from clinical and pre-clinical medicine, pharmacy and nursing throughout the 16-week programme.

Following four concept lectures on comprehensive healthcare and holistic approach to patients’ health problems, the students underwent small-group discussions with their facilitators. Each sub-group was given contact details and main diagnoses of real patients who had been invited and consented to participate in this programme. The facilitators guided the students on how to conduct a home visit and guided the students to identify the patients’ bio-psychosocial problems. The students learnt basic principles of management of the patients’ problems. The facilitators also guided the students to utilise the available resources in the community to optimise patients’ care.

In the 2011/2012 academic session, there were 202 medical students and 50 pharmacy students enrolled into this module. The students were divided into 17 groups of about 14–15 students each. These 12 groups were then subdivided into three smaller sub-groups. In view of the imbalance of the ratio between medical students and pharmacy students, only 25 sub-groups had the opportunity to work in inter-professional groups. Inter-professional sub-groups consisted of two pharmacy students and three medical students. Other sub-groups were comprised of all medical students. The pharmacy students were paired up together to avoid an imbalance in the group dynamics.

Students visited the assigned patients in small groups. Based on the information gathered and the small-group discussions, the students would identify a suitable community-based organisation relevant to their patient's needs and plan a learning visit to the organisation. At the end of the module, the students are required to present a family case study as a group, followed by a written family case report for assessment.

The module used formative assessment which included both individual and team performance. Supervisor's report, peer evaluation, reflective journals, case reports and case presentations were part of the assessment. An online Moodle website was utilised to allow students to download relevant course material as well as to submit their assignments. This website was also used by their facilitators to provide feedback to the students regarding their assignments. Each student was required to write a reflective journal on his or her role as an inter-professional team member in the care of the patient.

This programme was special as it offers early exposure to real patients in clinical and community settings. It also facilitated students to identify their own learning needs and problem solving skills.

To assess whether the students have benefited from the module through acquiring the knowledge and skills of collaborative teamwork, we have chosen to analyse the reflective journal as they documented the students’ experiences in IPE. Highly scored reflective journals (awarded 20 marks or more over a maximum of 25 marks) were selected for analysis. These reflective journals generally represent good quality reflections which go beyond descriptive writing and contained richer data. Reflective journals with lower marks were not selected as they did not demonstrate deep reflection and were assumed to be less accurate in representing what the students really learnt.

The researchers used thematic analysis to identify learning points expressed by the students. The reflective journals were read repeatedly by two of the researchers and coded individually. Also, any thoughts and opinion while interpreting the reflective journals were captured in the reflexive notes. The codes were then cross-checked by exchanging the notes. During the initial phase of coding, no specific theory or pre-determined codes were used. The themes were discussed and grouped into larger themes that formed meaningful categories. Diagrams and charting were used to help consolidating the themes. The process of consolidation was facilitated by re-visiting the reflexive notes written by the researchers.

## Results

There were a total of 252 reflective journals submitted by 202 medical students and 50 pharmacy students. Out of these, only 89 scored 20 and above from a maximum of 25 marks. Using purposive quota sampling, the reflective journals were selected to represent the distribution of the students based on faculty and grouping (See Table 1).

**Table 1:** Characteristics of the reflective journals

	*N*	Reflective journals which scored ≥20 (*n*)		Analysed (*n)*
Medical students	202	Uni-professional group*	52	14
		Inter-professional group**	24	7
Pharmacy students	50	Inter-professional group**	13	4

* Groups with only medical students.

** Groups with medical students and pharmacy students.

There were three major themes identified regarding what the students personally learnt from the module, which were: ‘Understanding the impact of illness and its relation to holistic care’, ‘Role of various healthcare professions’ and ‘Generic or soft skills for inter-professional collaboration’. There were no new themes arising after analysis of 25 reflective journals.

### Understanding of impact of illness and its relation to holistic care

Understanding the impact of illness led to an improved understanding of the importance of holistic care in patient management.

In clinical approach, we only look at Alzheimer's as a disease that threatens a patient physically, but in holistic approach, we get the chance to understand how the illness affects the patient emotionally and mentally as well. – Medical student, from an inter-professional group

As demonstrated in the reflection quoted above, the awareness of holistic care is important for better patient care. It also improved the capacity for the students to empathise with their patients as they struggled to cope with their illness. Understanding the multiple facets of the patients’ problems also helped the students realise that good quality healthcare cannot be achieved by one single profession.

### Role of various healthcare professions

A significant majority of the scripts revealed a new awareness regarding the need for and the roles of various healthcare professionals in patient management. Some of the medical students had never realised the importance of the allied health professionals in patient care. The visit to a related healthcare organisation was an eye opener for them.

Before that, I did not know that dieticians and diabetes educators also included in disease control. From the visit to PDM [Diabetes Association of Malaysia], I learnt that how each profession performs their job and how they work together. I ashamed of myself as I thought that doctor is the most important person in determining the recovery of a patient. – Medical student, from an inter-professional group

Previously, I thought that other professions have their own jobs. No one is depending on each other. But after the visit, it really enlightens me. Doctor is not god. We still have our limitation. That's why we need to depend on other professions to make the patients fully recover. – Medical student, from a uni-professional group

Students who had the benefit of belonging to a mixed group of medical and pharmacist students reflected that they gained from the skills of the other profession. Their previous perceptions towards the other profession were replaced by respect and appreciation.

I saw it obviously that there was a need between a doctor and a pharmacist in working life. – Medical student, from an inter-professional group

My medical group member do help me a lots. I am in charge to interview the patients’ family members. So, my medical group members do help me in preparing the questions for the interview like the type of question and the proper way to communicate with patient. – Pharmacy student, from an inter-professional group

Conversely, uni-professional groups consisting of only medical students realised that they were at a disadvantage. They found that having pharmacy students would help to complement their knowledge in assessing the patients.

Besides that, because we do not have any other courses in our group, especially pharmacist student, so we have difficulty so in pharmacology part we not really know about the medicine and also the drug interaction. – Medical student, from a uni-professional group

### Generic or soft skills for inter-professional collaboration

Although the theme for the reflective writing was based on inter-professional collaboration, many students reflected on how they learnt generic or soft skills during the running of the module. The students mentioned the importance of teamwork and communication in conducting the visits and completing their assignments.

Not only that, working with students from different course which are medic student really open up my eyes that it is not easy to achieve team work without good toleration, understanding, patient and communication skills… However, having the discussion with her make me realise that we are in the same group and whoever presenting whoever part is not important because whatever we do, we will be (re)presenting each other. – Pharmacy student, from an inter-professional group

The students had the opportunity to practice their leadership skills. Learning to take responsibility for the group's achievement would also be important to support future collaborative practice. The students learnt to work towards a common goal together, rather than on their own.

As a team leader in this inter-professional group, I need to compile all of their efforts and make the best slide presentation for our group. I did double check their parts that they were submitted to me. – Medical student, from a uni-professional group

I made every one contributed his/her professional knowledge to make up the short coming of others. Before blaming our group members, we should put ourselves in their shoes; it is not their fault to be lack of knowledge in other professional field. – Medical student, from an inter-professional group

Communication skills were mentioned frequently. The students found that good communication among the group members was important to facilitate teamwork.

So, in order to carry this task successfully, we had to contact each other, have a great discussion about it, and started on editing the report again with the reference on the required information for the report. This lead to improvement in communication and clear the misunderstanding and confusion among us and it changed the perception about each other. – Medical student, from a uni-professional group

For example, we have to be willing in interchanging information when needed so that our patient can receive the most appropriate treatment from us after considering various aspects in a holistic manner. – Medical student, from an inter-professional group

The home visit and experience in taking history from the patient also highlighted the importance of communication skills with patients and bedside manners. The students became aware of the influence of non-verbal skills as well as the importance of not using jargon in communicating with patients.

When I looked at other course mates, some of them just stood still in their position but did nothing, some of them started to chat among themselves. I started to think about the way of patients and volunteers might look us as a medical student. They might think that we are a bit self-centred and unfriendly. – Medical student, from a uni-professional group

But, we had some difficulty in explaining medical term to the patient, and I think it is one of the causes of difficulty in communication skills. – Medical student, from a uni-professional group

In view that Malaysia has a diverse, multi-cultural and multi-ethnic population, language barriers can be a challenge for healthcare professionals. The students had to rely on their group members or a relative to translate in order for them to gather history from the patient. This may expose them to cultural awareness in communicating with patients.

Luckily, two of my group members can spoke Mandarin and helped translating any important information to us. – Pharmacy student, from an inter-professional group

I was unable to interpret the information given by the caretaker as he was comfortable communicating in Malay. – Medical student, from a uni-professional group

## Discussion

The feedback and reflective journals demonstrated that early exposure to real-life patients in the community setting helped to improve the students’ awareness regarding the impact of health problems to the patients, their family and the community. It was a valuable experience for both medical and pharmacy students as they learnt from their assigned patients, as well as the community-based healthcare organisations. Past studies have also demonstrated that students who were given early clinical exposure had better empathy towards their patients and improved their understanding of clinical practice (1).

Such exposure also offers them the benefit of learning how to apply a bio-psychosocial approach in patient assessment, as well as multi-disciplinary management of the patients’ problems (6). The ability to facilitate and coordinate multi-disciplinary care for the patient is an essential skill in the management of chronic diseases.

IPE has been identified as a strategy that is pivotal in improving healthcare worldwide (2). This is in tandem with the need for good collaborative practices in order to deliver good quality healthcare to patients. IPE elements have been implemented in many medical schools in the West, with great success and positive outcomes (7).

The CHC module is the first initiative to incorporate elements of IPE into the undergraduate medical curriculum in this university. Although it has yet to completely fulfil the criteria for IPE, it has been structured to provide shared learning opportunities. The objectives of the course would not be achieved successfully, if the students were to study in a fully uni-professional environment. For example, pharmacy students contributed their expertise in issues related to patient medication, whereas medical students contributed their knowledge regarding clinical history and disease pathophysiology. Expertise from both backgrounds was necessary in providing patient education.

The themes obtained from the students’ reflective writing illustrated the shared learning acquired by both groups of students and demonstrated their valuable experience in IPE. The reflective writing revealed that the module was an eye opener for them with regards to the roles of other healthcare professionals in delivery of healthcare to patients. This had also been shown in medical schools elsewhere (8, 9).

The shared learning experiences also altered their pre-conceived perceptions and attitudes between the medical and pharmacy students. They became aware of the limitations of their own professions and developed respect towards other healthcare professions. They realised that the provision of comprehensive healthcare requires the contribution of an inter-professional team. This is supported by other studies which studied the attitudes of healthcare students before, during and after an IPE programme (8, 10).

Certain soft skills are vital to the success of collaborative practice. These include communication skills and teamwork (2). The ability to communicate well between the various professions as well as within their own professions is required for successful collaborative practice. Thus, it is one of the learning outcomes for inter-professional learning. The ability to work as a team is also vital. Members of the team should be able to set aside pre-conceived attitudes towards others and be willing to work together under the team leader. The students’ reflective writings clearly talked about the acquisition and appreciation of communication skills and teamwork. Therefore, the shared learning experiences within the CHC module helped to prepare the students for future collaborative practice.

There were several challenges worthy of mention in the implementation of this module. The limited number of pharmacy students versus the medical students led to unequal distribution, and a significant number of groups were uni-professional, consisting of only medical students. This may be overcome should other undergraduate healthcare professions be included into this module in the future.

Another challenge was the difficulty in scheduling the programme as different undergraduate programmes had different schedules. Scheduling difficulties were also faced by other medical schools implementing inter-professional initiative (7). A concerted effort between participating faculties is required in order to smooth out these logistic barriers in the implementation of the module.

This study is limited by the small number of reflective journals selected for analysis. However, they represented the depth of their learning experiences well. The themes identified from these 25 reflective journals were recurring and hence, data saturation was achieved. Analysing the remaining reflective journals, with lower scores, may not add new information because their scores could be due to the students’ poor command of English rather than their actual learning experiences. English was not the first language for these students.

## Conclusion

To sum up, the CHC module was an effective introduction of IPE initiatives for healthcare students in this university. Overcoming the challenges faced during the organisation of the module would require concerted effort from the faculty members. This included strengthening the inter-professional learning elements for the module. Ongoing improvements and innovation to the module is hoped to bring out the full potential of this module in enriching the learning experience of the students.
